# A numerical treatment of MHD radiative flow of Micropolar nanofluid with homogeneous-heterogeneous reactions past a nonlinear stretched surface

**DOI:** 10.1038/s41598-018-30965-x

**Published:** 2018-08-20

**Authors:** Dianchen Lu, M. Ramzan, Shafiq Ahmad, Jae Dong Chung, Umer Farooq

**Affiliations:** 1 Department of Mathematics, Faculty of Science, Jiangsu University, Zhenjiang, Jiangsu China; 2 Department of Computer Science, Bahria University, Islamabad Campus, Islamabad, 44000 Pakistan; 3 Department of Mathematics, Quaid-I-Azam University, Islamabad, 44000 Pakistan; 4 Department of Mechanical Engineering, Sejong University, Seoul, 143-747 Korea; 5 Department of Mathematics, COMSATS University, Park road, Tarlai Kalan, Islamabad, 45550 Pakistan

## Abstract

The impact of nonlinear thermal radiation in the flow of micropolar nanofluid past a nonlinear vertically stretching surface is investigated. The electrically conducting fluid is under the influence of magnetohydrodynamics, heat generation/absorption and mixed convection in the presence of convective boundary condition. The system of differential equations is solved numerically using the bvp4c function of MATLAB. To authenticate our results, two comparisons with already studied problems are also conducted and an excellent concurrence is found; hence reliable results are being presented. Complete deliberation for magnetite nanofluid with Ferric Oxide (Fe_3_O_4_) nanoparticles in the water-based micropolar nanofluid is also given to depict some stimulating phenomena. The effect of assorted parameters on velocity, homogeneous-heterogeneous reactions, temperature and micropolar velocity profiles are discussed and examined graphically. Moreover, graphical illustrations for the Nusselt number and Skin friction are given for sundry flow parameters. It is examined that temperature distribution and its associated boundary layer thickness increase for mounting values of the magnetic parameter. Additionally, it is detected that the Nusselt number decays when we increase the values of the Biot number.

## Introduction

Flows over stretched surfaces have various engineering and industrial applications like the extrusion of plastic sheets, extraction of polymer, glass blowing, drawing of wires, paper production and rubber sheets^1^. The pioneering work of flow of Newtonian fluid over a moving rigid surface by Sakiadis^2^ has motivated the follower scientists and researchers to reveal more thought-provoking ideas in this core area. Sakiadis’s idea was improved by Crane^3^ who considered a stretching sheet instead of a rigid surface. This was followed by an excellent research article by Gupta and Gupta^4^ who studied the effect of heat transfer on a viscid fluid past a stretching sheet. Later, Chakrabarti and Gupta^5^ improved the work of Gupta and Gupta^4^ by analyzing the flow of viscid fluid past a stretching sheet in the presence of magnetohydrodynamics. Devi and Ganga^6^ discussed dissipation impacts on nonlinear flow with magnetohydrodynamics past a permeable medium. Considering the importance of flows over stretched surfaces, scientists and researchers are encouraged even today to look for new ideas. Some salient contributions in this regard include a study by Hsiao^7^ who discussed the impacts of Ohmic dissipation and MHD on the flow of viscoelastic fluid through a stretched sheet. Ibrahim *et al*.^8^ deliberated the magnetohydrodynamics nanofluid flow over a stretching surface. Lin *et al*.^9^ inspected the Pseudoplastic flow of nanofluid past a time-dependent stretching surface under the influence of variable thermal conductivity, viscous dissipation, and Joule heating. The nanofluid flow over the stretching sheet, with the effects of stagnation point, heat source/sink and electro magnetohydrodynamic with slip boundary condition, was analyzed by Hsiao^10^. Hayat *et al*.^11^ found the series solution for the third-grade fluid flow with an impact of chemical reaction past an unsteady stretching surface. Malvandi *et al*.^12^ obtained the numerical solution of the flow of variable nanofluid in the neighborhood of a stagnation point with Navier’s slip condition. Numerical solution of flow of MHD nanofluid past a shrinking/stretching surface in a spongy medium near a stagnation point was discussed by Khalili *et al*.^13^. The flow of Jeffrey fluid over a linearly stretching sheet was examined by Hayat *et al*.^14^. Hayat *et al*.^15^ also analyzed the Powell Eyring fluid flow past a stretched surface mounted at an angle with effects radiation and non-uniform sink/source. Ahmadi *et al*.^16^ inspected a detailed study of nanofluid flow past an unsteady extended surface. Chen *et al*.^17^ found the numerical solution of Maxwell fluid flow past a time-dependent stretched surface. Hua and Su^18^ studied the time-dependent flow over a stretched surface in a moving fluid with impacts of frictional and Ohmic heating. Hayat *et al*.^19^ explained the flow of viscous fluid past an exponential stretched surface with slip condition and magnetohydrodynamics. Waqas *et al*.^20^ found numerical solution of the micropolar fluid flow with effects of mixed convection, magnetohydrodynamics and convective boundary conditions past a nonlinear stretched surface. Lu *et al*.^21^ debated the flow of magneto-hydrodynamic Carreau nanofluid flow numerically with impacts of nonlinear thermal radiation with zero mass flux condition past a radially stretching sheet.

Flows involving effects of the chemical reactions have gained the attraction of the researchers and scientists. Applications of these flows in various processes may include the production of ceramics and polymers, chemical processing, fibrous insulations, water and air pollutions and molecular diffusion of species. Chemical reactions are labeled as homogeneous- heterogeneous (h & h) reactions. The domain in the case of heterogeneous reactions is restricted however it covers the entire phase uniformly. Combustion and catalysis cover both h & h reactions. Moreover, the heterogeneous catalyst is found in the solid phase while homogeneous catalyst exists in gaseous and liquid phases. Recent investigations involving the impacts of h & h reactions include a study by Imran *et al*.^22^ who discussed the flow of Casson fluid numerically with effects of h & h reactions with magnetohydrodynamics and viscous dissipation. Hayat *et al*.^23^ examined analytical solution of 2D micropolar fluid with h & h reactions and magnetohydrodynamics. The flow of 3D Oldroyd-B fluid flow with nonlinear thermal radiation and homogeneous-heterogeneous (h-h) reactions is discussed by Lu *et al*.^24^. Abbas and Sheikh^25^ numerically found the solution of Ferrofluid with nonlinear slip condition and h & h reactions. Nadeem *et al*.^26^ explored the influence of magnetic dipole numerically past a stretched cylinder with h & h reactions. Raju *et al*.^27^ found dual solutions of Jeffrey fluid flow numerically with the impact of h & h reactions. Imad *et al*.^28^ examined the Prandtl fluid flow with h & h reactions past a linearly stretched surface.

During the last few years, the heat transport phenomenon in fluid flows got broad consideration owing to its varied applications in engineering and technology. Nanofluids are comprised of nanometer-sized particles, named as nanoparticles, and some usual base fluids. The nanoparticles used in nanofluids are characteristically composed of oxides, metals, carbon nanotubes, or carbides. The concept of nanofluids was first time given by Choi^29^ Later, Keblinski *et al*.^30^ experimentally and numerically proved that the thermal conductivity of nanofluids is prominent in comparison to the ordinary base fluids. Consequently, usage of nanofluids in engineering applications like in domestic refrigerators, nuclear reactors, chiller, heat exchangers, engine cooling and vehicle thermal management has immensely increased. More about applications of nanofluids may be found in^31^. Our intention here is to report different latest studies highlighting varied aspects of nanofluids. These may include an investigation by Nadeem and Noor^32^ who investigated the impact of Cattaneo-Christov mass and heat flux in a nanofluid implanted in a spongy medium. Nadeem and Lee^33^ used an exponentially stretching surface to deliberate the flow of nanofluid. Sheikholeslami^34^ described the ethylene glycol nanofluid flow in the presence of thermal radiation past a porous enclosure. Nadeem *et al*.^35^ considered the influence of mass and heat transfer on peristaltic flow of a nanofluid between eccentric cylinders. Sheikholeslami^36^ used complex geometry to discuss the impact of the electric field on the hydrothermal conduct of nanofluid. In the presence of porous medium Raju and Sandeep^37^ investigated radiative magnetohydrodynamic Jeffrey nanofluid flow past a cone with chemical reaction.

A literature survey indicates that abundant studies are available discussing the flow of micropolar fluid flow with different geometries. Comparatively less work is done with micropolar nanofluid flow and as far as our knowledge is concerned no study so far is conducted for the flow of micropolar nanofluid with h-h reactions over a nonlinear vertically stretched sheet. Additional features of nonlinear thermal radiation and heat absorption/generation coefficient are also added characteristics towards its novelty. Numerical solution of the transformed equations is gained by utilizing the built-in function bvp4c. A complete parametric investigation is conducted using the graphical depiction of different controlling flow parameters on axial velocity, h-h reactions, temperature, microrotation velocity, and concentration profile. Additionally, the local Nusselt and friction factor numbers are graphed and dissected for increasing dynamical parameters. A comparison to a previously done study is also conducted to validate our presented results. An excellent alignment is obtained in this regard which proves that our results are indubitable.

## Theory and Flow Field Analysis

**Figure 1:**
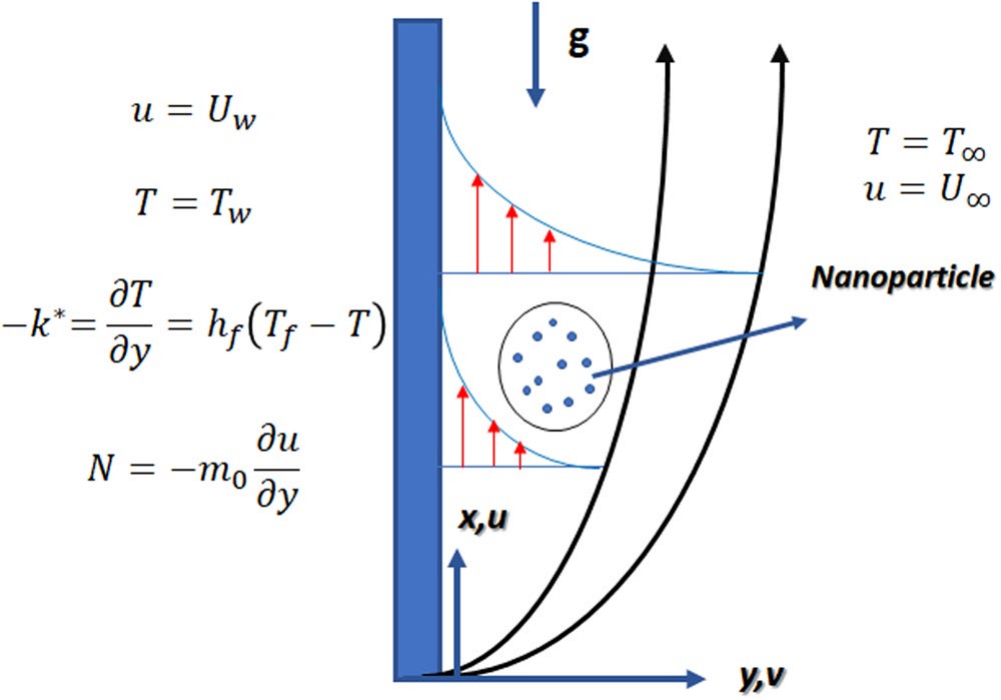
Schematic diagram of the model.

We assume an incompressible, steady two-dimensional boundary layer flow of micropolar nanofluid past a nonlinear vertically stretching surface with stretching velocity *u*(*x*) = *U*_*w*_(*x*) = *cx*^*n*^ along *x*-*axis*, where *c* is a constant. The flow is under the impacts of the nonlinear magnetic field, nonlinear thermal radiation, heat absorption/generation coefficient, and h & h reactions. The strength of the magnetic field is taken as *B*(*x*) = *B*_0_*x*^(*n*−1)/2^ acting in a normal direction to *y*-*axis*. We also assume that in qusient micropolar nanofluid both base fluid (water) and nanoparticles (Fe_3_O_4)_ are in thermal equilibrium and there is no chance of slip occurrence. The temperature at the surface is *T*_*w*_ where the ambient temperature is taken as *T*_∞_ as *y*→∞. Schematic diagram of the flow problem is depicted in Fig. 1.

The model for homogeneous (isothermal cubic autocatalytic) and heterogeneous reactions with two chemical species A* and B* was proposed by Merkin^38^ and Chaudhary and Merkin^39,40^ is given by

1 $${A}^{\ast }+2{B}^{\ast }\to 3{B}^{\ast },\,\,\,{\rm{rate}}={k}_{c}a{b}^{2},$$

2 $${A}^{\ast }\to {B}^{\ast },\,\,\,{\rm{rate}}={k}_{s}a,$$

where the concentration of chemical species B^*^ and A^*^ are given by *b* and *a* and *k*_*i*_,(*i* = *c*, *s*) are the rate quantities. Both reaction forms are considered as isothermal. Applying the boundary layer approximation, the continuity, micropolar, energy and concentration equations can be stated as follows:

3 $$\frac{\partial u}{\partial x}+\frac{\partial v}{\partial y}=0,$$

4 $$u\frac{\partial u}{\partial x}+v\frac{\partial u}{\partial y}=({\upsilon }_{nf}+\frac{\kappa }{{\rho }_{nf}})\frac{{\partial }^{2}u}{\partial {y}^{2}}+\frac{\kappa }{{\rho }_{nf}}\frac{\partial N}{\partial y}+{g}_{1}\beta (T-{T}_{\infty })-\frac{{\sigma }_{nf}{B}^{2}(x)}{{\rho }_{nf}}u,$$

5 $$u\frac{\partial N}{\partial x}+v\frac{\partial N}{\partial y}=\frac{{y}^{\ast }}{{\rho }_{nf}j}\frac{{\partial }^{2}N}{\partial {y}^{2}}-\frac{\kappa }{{\rho }_{nf}j}(2N+\frac{\partial u}{\partial y}),$$

6 $$u\frac{\partial T}{\partial x}+v\frac{\partial T}{\partial y}={\alpha }_{nf}\frac{{\partial }^{2}T}{\partial {y}^{2}}-\frac{1}{{(\rho {c}_{p})}_{nf}}\frac{\partial {q}_{r}}{\partial y}+\frac{{Q}_{0}}{{(\rho {c}_{p})}_{nf}}(T-{T}_{\infty }),$$

7 $$u\frac{\partial a}{\partial x}+v\frac{\partial a}{\partial y}={D}_{A}\frac{{\partial }^{2}a}{\partial {y}^{2}}-{k}_{c}a{b}^{2},$$

8 $$u\frac{\partial b}{\partial x}+v\frac{\partial b}{\partial y}={D}_{B}\frac{{\partial }^{2}b}{\partial {y}^{2}}+{k}_{c}a{b}^{2}.$$

These equations are supported by the following boundary conditions

9 $$\begin{array}{c}{u|}_{y=0}={U}_{w}(x)=c{x}^{n},\,{v|}_{y=0}=0,\,N=-\,{m}_{0}{\frac{\partial u}{\partial y}|}_{y=0},\\ {-{{k}_{1}}^{\ast }\frac{\partial T}{\partial y}|}_{y=0}={h}_{f}({T}_{f}-T),\,{D}_{A}{\frac{\partial a}{\partial y}|}_{y=0}={k}_{s}a,\,{D}_{B}{\frac{\partial b}{\partial y}|}_{y=0}=-\,{k}_{s}a,\\ \,\,\,{u|}_{y\to \infty }\to 0,\,{N|}_{y\to \infty }\to 0,\,{T|}_{y\to \infty }\to {T}_{\infty },\,{a|}_{y\to \infty }\to {a}_{0},\,{b|}_{y\to \infty }\to 0,\end{array}$$

where ${y}^{\ast }=j({\mu }_{nf}+\frac{\kappa }{2})$ shows the spin gradient viscosity. The symbols *g*_1_, *β*, *κ*, *B*(*x*), *N*, *j*, *Q*_0_, (*D*_*A*_, *D*_*B*_), (*k*_*c*_, *k*_*s*_), *m*_0_, k^*^, *h*_*f*_, *T*_*f*_, *n* are the gravitational acceleration, thermal expansion coefficient, magnetic parameter, coefficient of volumetric thermal expansion, microrotation velocity, micro inertia, the variable volumetric rate of heat source, diffusion coefficients, rate constants, boundary parameter, mean absorption coefficient, convective heat transfer coefficient, convective fluid temperature, and power law index respectively. It is pertinent to mention that the value of *n* directly relates to the shape of the surface. For *n* = 1, the surface is flat, for *n* > 1, the surface is inner concave and for *n* < 1, the surface is outer convex. Similarly, the values *n* can also predict the motion of the fluid. The values *n* = 1, *n* < 1, and *n* > 1, represent the linear motion of the fluid with constant velocity decelerated motion and accelerated motion respectively. In Eq. (6), nonlinear thermal radiation heat flux *q*_*r*_ via Rosseland’s approximation is given by

10 $${q}_{r}=\frac{4{\sigma }^{\ast }}{3{k}^{\ast }}\frac{\partial {T}^{4}}{\partial y}=\frac{16{\sigma }^{\ast }{T}^{3}}{3{k}^{\ast }}\frac{\partial T}{\partial y}$$

The values of the specific heat *C*_*p*_, the density *ρ*, the thermal conductivity *K*_1_ and the electric conductivity *σ* of the base fluid (water) and the magnetite nanoparticles (Fe_3_O_4_) (water) are given in Table 1.

**Table 1:** Thermo-physical characteristics of the base fluid (water) and nanoparticles (Fe_3_O_4_).

Physical properties	Base fluid (water)	Fe_3_O_4_
C_p_ (*J/kg K*)	4179.00	670
*ρ* (*kg/m*^3^)	997.100	5180
K_1_ (*W/mK*)	0.61300	9.7
*σ* (*Ωm*)	1.4700	1163.1

The mathematical form for the thermophysical properties are

11 $$\begin{array}{rcl}{\mu }_{nf} & = & \frac{{\mu }_{f}}{{(1-{\varphi })}^{2.5}},\,\frac{{\rho }_{nf}}{{\rho }_{f}}=1-{\varphi }+{\varphi }\frac{{\rho }_{s}}{{\rho }_{f}},\\ \frac{{\sigma }_{nf}}{{\sigma }_{f}} & = & 1-\frac{3\varphi [1-\tfrac{{\sigma }_{s}}{{\sigma }_{f}}]}{[2+\tfrac{{\sigma }_{s}}{{\sigma }_{f}}]+\varphi [1-\tfrac{{\sigma }_{s}}{{\sigma }_{f}}]},\\ {\alpha }_{nf} & = & \frac{{k}_{nf}}{{(\rho {C}_{p})}_{nf}},\,\frac{{(\rho {C}_{p})}_{nf}}{{(\rho {C}_{p})}_{f}}=(1-{\varphi })+{\varphi }\frac{{(\rho {C}_{p})}_{s}}{{(\rho {C}_{p})}_{f}},\\ \frac{{k}_{nf}}{{k}_{f}} & = & \frac{(2{k}_{f}+{k}_{s})-2{\varphi }({k}_{f}-{k}_{s})}{(2{k}_{f}+{k}_{s})+{\varphi }({k}_{f}-{k}_{s})}.\end{array}$$

### Similarity transformation

Using the following transformations

12 $$\begin{array}{c}\psi =f(\eta )\sqrt{\frac{2{\nu }_{f}x{u}_{e}(x)}{n+1}},\,\eta =y\sqrt{\frac{(n+1){u}_{e}(x)}{2x{\nu }_{f}}},\,N=c{x}^{n}\sqrt{\frac{(n+1){u}_{e}(x)}{2x{\nu }_{f}}}g(\eta ),\\ u=c{x}^{n}f^{\prime} (\eta ),\,v=-\sqrt{\frac{(n+1){\nu }_{f}c{x}^{n-1}}{2}}\{f(\eta )+(\frac{n-1}{n+1})\eta f^{\prime} (\eta )\},\\ \,\,{T}_{\infty }+({T}_{w}-T)\theta (\eta )=T,\,a={a}_{0}h,\,b={a}_{0}\phi .\end{array}$$

The requirement of continuity equation is inevitably satisfied, and Eqs (4) to (8) become

13 $$\begin{array}{c}(\frac{1}{{(1-{\varphi })}^{2.5}(1-{\varphi }+{\varphi }\tfrac{{\rho }_{s}}{{\rho }_{f}})}+\frac{K}{(1-{\varphi }+{\varphi }\tfrac{{\rho }_{s}}{{\rho }_{f}})})\,f\prime\prime\prime +ff^{\prime\prime} -(\frac{2n}{n+1})\,{f}^{^{\prime} 2}+\frac{K}{(1-{\varphi }+{\varphi }\tfrac{{\rho }_{s}}{{\rho }_{f}})}g^{\prime} \\ \,+\,\frac{2}{n+1}(\lambda \theta -\frac{M({\sigma }_{nf}/{\sigma }_{f})}{(1-{\varphi }+{\varphi }\tfrac{{\rho }_{s}}{{\rho }_{f}})}f^{\prime} )=0,\end{array}$$

14 $$\begin{array}{c}(\frac{1}{{(1-{\varphi })}^{2.5}(1-{\varphi }+{\varphi }\tfrac{{\rho }_{s}}{{\rho }_{f}})}+\frac{K}{2(1-{\varphi }+{\varphi }\tfrac{{\rho }_{s}}{{\rho }_{f}})})g^{\prime\prime} +g^{\prime} f-(\frac{3n-1}{n+1})gf^{\prime} -\frac{2K}{(n+1)(1-{\varphi }+{\varphi }\tfrac{{\rho }_{s}}{{\rho }_{f}})}\\ \,(2g+f^{\prime\prime} )=0,\end{array}$$

15 $$\begin{array}{c}(\frac{{k}_{nf}}{{k}_{f}}+{R}_{d}{(1+({\theta }_{w}-1)\theta )}^{3})\theta ^{\prime\prime} +\Pr (1-\varphi +\varphi \frac{{(\rho {C}_{p})}_{s}}{{(\rho {C}_{p})}_{f}})(f\theta ^{\prime} -\frac{2\gamma }{n+1}\theta )\\ \,+\,3{R}_{d}({\theta }_{w}-1){(1+({\theta }_{w}-1)\theta )}^{2}{\theta ^{\prime} }^{2}=0,\end{array}$$

16 $$\frac{1}{{S}_{c}}h^{\prime\prime} +fh^{\prime} -\frac{2{k}_{1}}{n+1}h{\phi }^{2}=0,$$

17 $$\frac{\delta }{{S}_{c}}\phi ^{\prime\prime} +f\phi ^{\prime} +\frac{2{k}_{1}}{n+1}h{\phi }^{2}=0,$$

and the boundary conditions in Eq. (9) yield the following form

18 $$\begin{array}{rcl}f(\eta ) & = & 0,\,f^{\prime} (\eta )=1,\,\theta \text{'}(\eta )=-\,{B}_{i}(1-\theta (\eta )),\,g(\eta )=-\,{m}_{0}f^{\prime} (\eta ),\\ h^{\prime} (\eta ) & = & {k}_{2}h(\eta ),\,\delta \phi ^{\prime} (\eta )=-\,{k}_{2}h(\eta )\,{\rm{as}}\,\eta =0,\\ \theta (\eta ) & = & 0,\,f^{\prime} (\eta )=0,\,g(\eta )=0,\,\phi (\eta )=0,\,h(\eta )=1,\,{\rm{at}}\,\eta \to \infty ,\end{array}$$

where prime designate the derivative with respect to *η*. The parameters *k*_2_, *M*, *K*, *R*_*d*_, *γ*, *k*_1_, *S*_*c*_, *δ*, *B*_*i*_, *λ* represent the strength of heterogeneous reaction, magnetic parameter, micropolar parameter, radiation parameter, heat generation parameter, the strength of homogeneous reaction, Schmidt number, the ratio of diffusion coefficient, Biot number and mixed convection parameter respectively are defined as follows

19 $$\begin{array}{c}\lambda =\frac{G{r}_{x}}{{\mathrm{Re}}_{x}^{2}},\,K=\frac{\kappa }{{\mu }_{f}},\,M=\frac{{\sigma }_{f}{B}^{2}(x)}{c{\rho }_{f}},\,{\rm{\Pr }}=\frac{{\nu }_{f}}{{\alpha }_{f}},\,{R}_{d}=\frac{16{\sigma }^{\ast }{T}_{\infty }^{3}}{3k{k}^{\ast }},\\ {\mathrm{Re}}_{x}=\frac{{u}_{w}x}{{\nu }_{f}},\,G{r}_{x}=\frac{{g}_{1}\beta ({T}_{w}-{T}_{\infty }){x}^{3}}{{\nu }_{f}^{2}},\,\gamma =\frac{{Q}_{0}}{c{(\rho {C}_{p})}_{f}},\,{S}_{c}=\frac{{\nu }_{f}}{{D}_{A}},\\ {k}_{1}=\frac{{{a}_{0}}^{2}x{k}_{c}}{{U}_{w}},\,{k}_{2}=\frac{{k}_{s}}{{D}_{A}}\sqrt{\frac{x{\nu }_{f}}{{U}_{w}}},\,{B}_{i}=\frac{{h}_{f}}{{k}_{1}^{\ast }}\sqrt{\frac{x{\nu }_{f}}{{U}_{w}}},\,\delta =\frac{{D}_{A}}{{D}_{B}}.\end{array}$$

In general, the chemical species *A* and *B* won’t be identical, nevertheless, we can expect that these will be equivalent in size^38^. Here, we suppose that the diffusion species coefficients *D*_*B*_ and *D*_*A*_ are identical, *i.e., δ* = 1, thus we have

20 $$\phi (\eta )+h(\eta )=1.$$

Now applying the above property, Eqs (16) and (17) and their corresponding boundary condition take the form

21 $$\frac{1}{{S}_{c}}h^{\prime\prime} +fh^{\prime} -\,\frac{2{k}_{1}}{n+1}h{(1-h)}^{2}=0,$$

22 $$h^{\prime} (0)={k}_{2}h(0),\,h(\infty )\to 1.$$

### Friction factor and local Nusselt number

The dimensional forms of the *C*_*f*_ (skin friction coefficient) and *Nu*_*x*_ (the local Nusselt number) are categorized as

23 $${C}_{f}=\frac{2{\tau }_{w}}{{\rho }_{f}{u}_{w}^{2}},\,\,N{u}_{x}=\frac{x{q}_{w}}{{k}_{1}^{\ast }({T}_{f}-{T}_{\infty })}.$$

Now the surface heat flux (*q*_*w*_) and surface shear stress (*τ*_*w*_) are assumed as:

24 $${\tau }_{w}={(\frac{\partial u}{\partial y}(\kappa +{\mu }_{nf})+\kappa N)}_{y=o},\,{q}_{w}=-\,{(\frac{\partial T}{\partial y})}_{y=0}{k}_{nf}+{({q}_{r})}_{y=0}.$$

Using Eqs (12) and (24) in Eq. (23), we acquire

25 $$\begin{array}{rcl}{{{\rm{Re}}}_{x}}^{1/2}{C}_{f} & = & \sqrt{\frac{n+1}{2}}(\frac{1}{{(1-\phi )}^{2.5}}+(1-{m}_{0})K)f^{\prime\prime} (0),\\ {{{\rm{Re}}}_{x}}^{-1/2}N{u}_{x} & = & -\frac{{k}_{nf}}{{k}_{f}}\sqrt{\frac{n+1}{2}}\theta ^{\prime} (0),\end{array}$$

where ${\mathrm{Re}}_{x}=\frac{{u}_{w}x}{{\nu }_{f}}$ is the local Reynolds number.

## Results and Discussion

The numerical solution, of Eqs (13, 14, 15, 21) with associated boundary conditions Eqs (18) and (22), is obtained using MATLAB built-in function bvp4c. For this purpose, initially, we have converted the third and second order differential equations to the first-order ordinary differential equations using new parameters. The built-in function bvp4c requires an initial supposition and the tolerance for the current problem is given as 10^−7^. Considering the values of the involved parameters, we have used suitable finite values of *η*→∞, as *η* = *η*_∞_ = 3 *to* 6. For the computation of the numerical solution, we have selected the following fixed values of involved parameters *M* = 0.1, Pr = 6.2, *K* = 0.5, *ϕ* = 0.1, *m*_0_ = 0.5, *B*_*i*_ = 0.5, *S*_*c*_ = 3.

Tables 2 and 3 are erected numerically to validate our presented results by fixing values of different parameters. It is found that all obtained results are in good alignment to those given in Reddy^41^ and Cortell^42^.

**Table 2:** Comparison of −*f* ″(0) with^41^ for numerous values of *n* and *M* when $\Pr =0.7,$*ϕ* = 0.0.

*n*	*M*	−*f* ″(0)	
		Reddy^41^	Present result
1	0.5	0.865956	0.86595
	01	1.097058	1.09705
	02	1.753714	1.75371
	03	2.744580	2.74458
2	0.5	0.950290	0.95029
	01	1.101523	1.038900
	02	1.572680	1.57268
	03	2.429416	2.42941

**Table 3:** Comparison of −*f* ″(0) with^42^ for various values of *n* in the absence of the micropolar nanofluid and h-h reactions when *M* = 0 = *ϕ* = *λ*.

*n*	−*f* ″(0)	
	Cortell^42^	Present result
0.0	0.627547	0.627547
0.2	0.766758	0.766758
0.5	0.889477	0.889477
0.75	0.953786	0.953786
1.0	1.0	1.0
1.5	1.061587	1.061587
3.0	1.148588	1.148588

**Figure 2:**
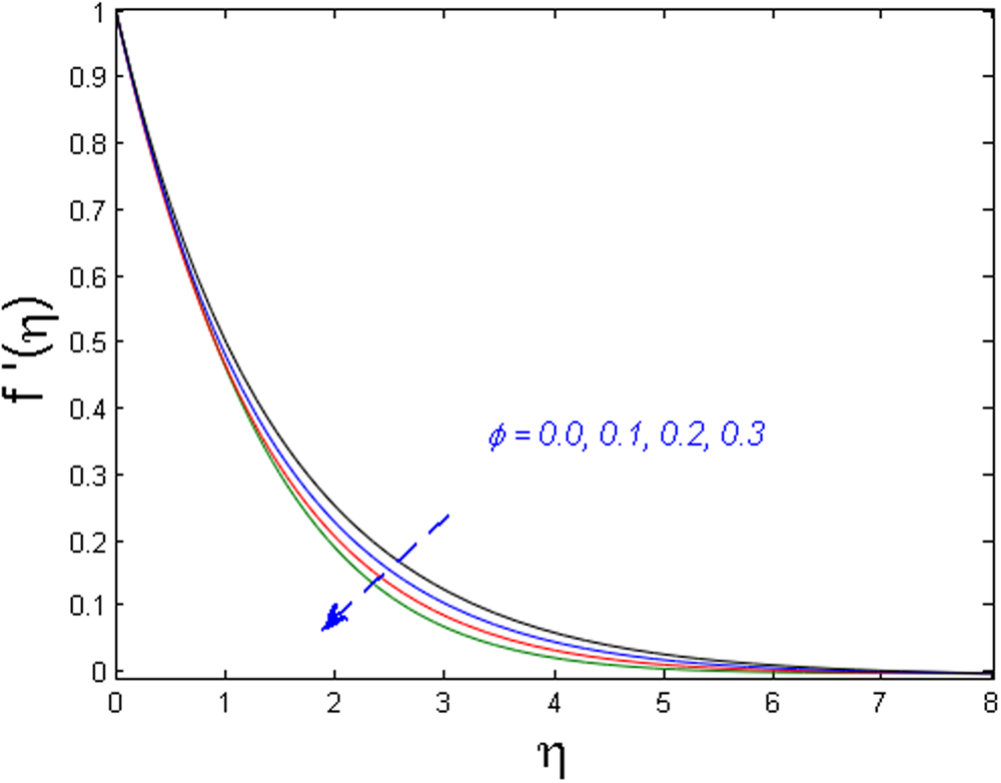
Effect of *ϕ* on *f*′(*η*).

**Figure 3:**
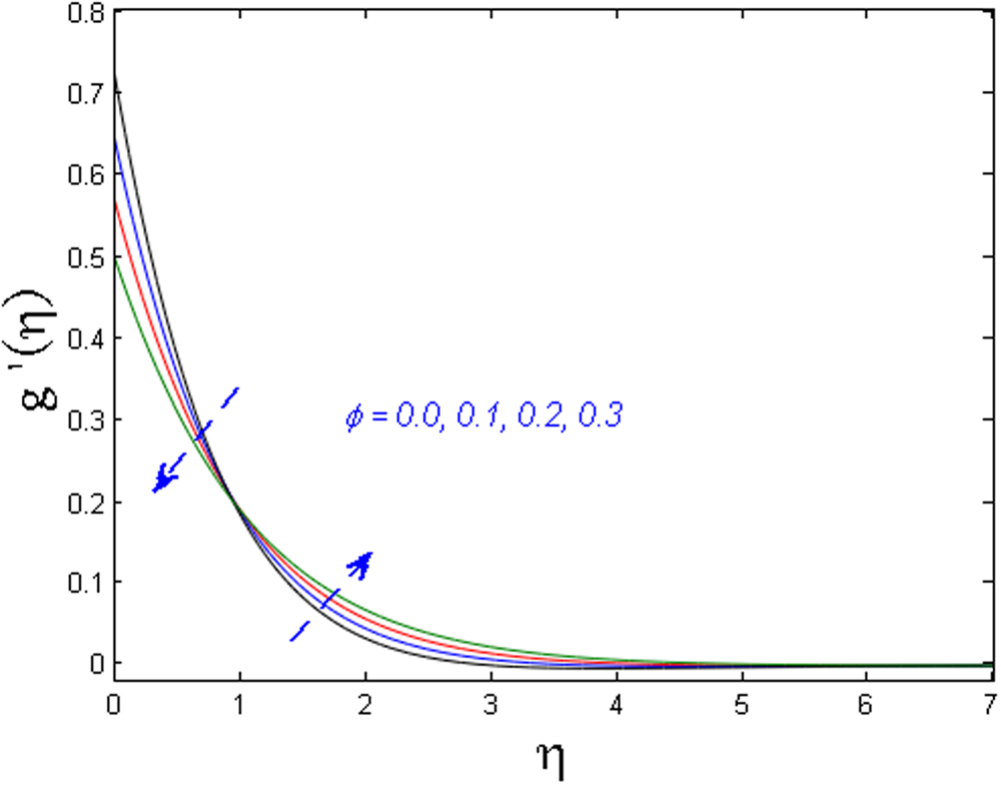
Effect of *ϕ* on *g*′(*η*).

**Figure 4:**
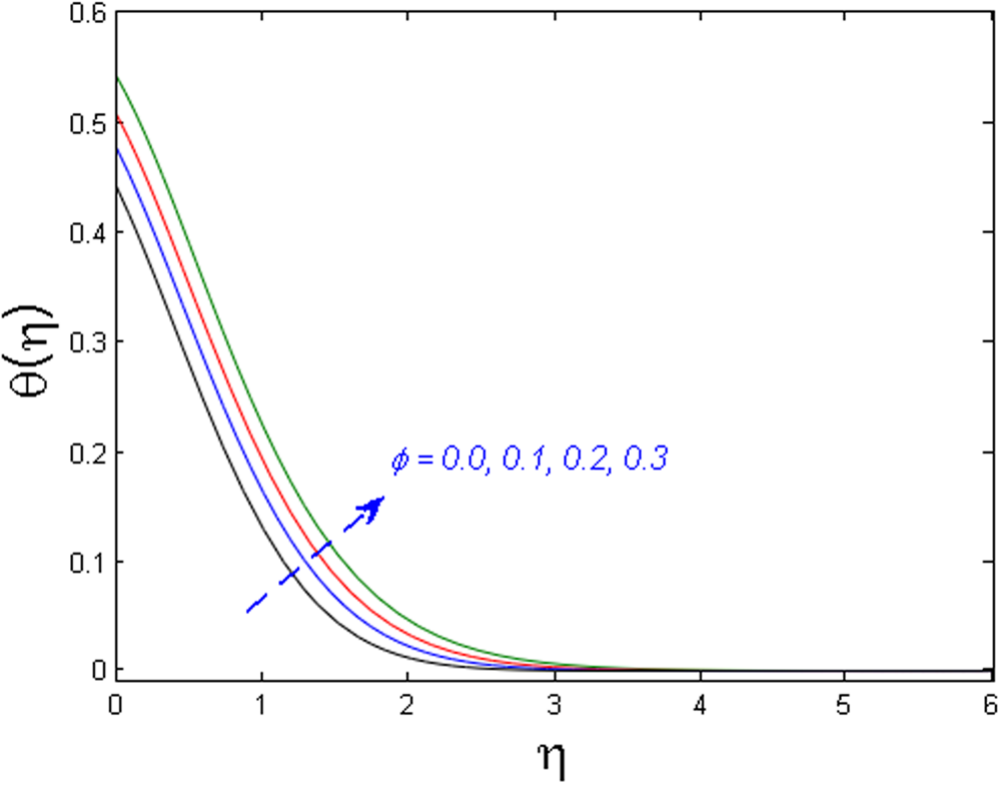
Effect of *ϕ* on *θ*(*η*).

The impact of solid volume fraction *ϕ* on axial velocity, microrotation and temperature distributions is depicted in Figs 2–4. Figure 2 shows that the axial velocity profile decline versus increasing values of volume fraction. The effect of *ϕ* on microrotation profile is given in Fig. 3. It is perceived that the microrotation profile decays near and grows far away from the boundary layer and satisfies it asymptotically. While the temperature distribution diminishes, and its associated thermal boundary layer thickness augments when values of *ϕ* are increased as given in Fig. 4.

**Figure 5:**
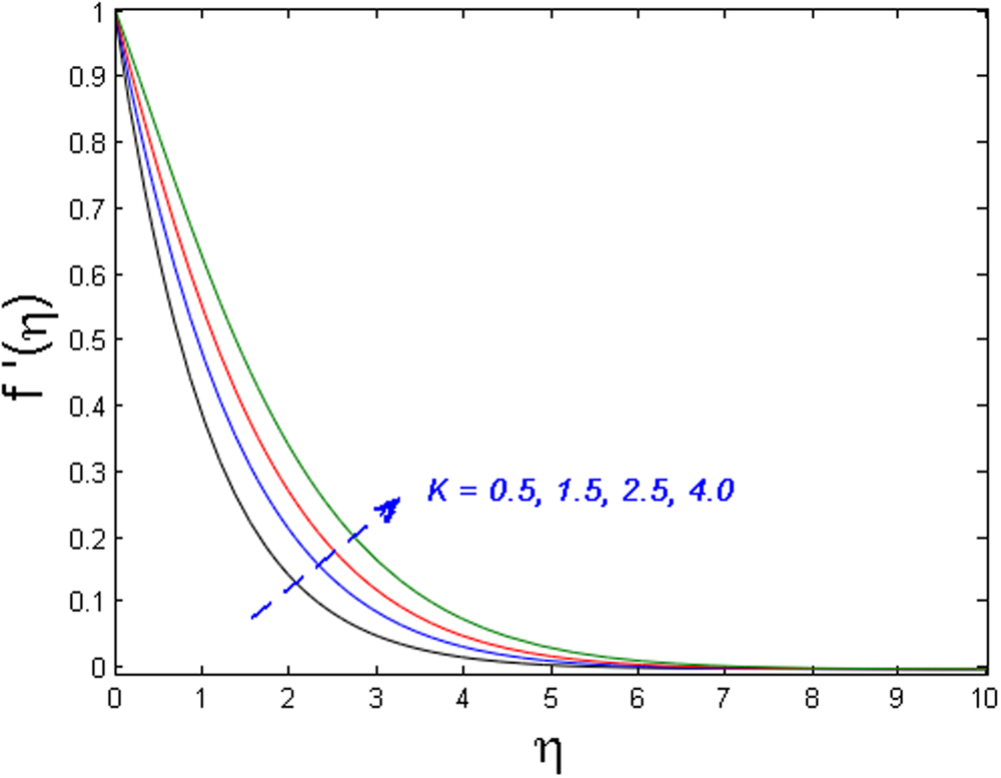
Effect of *K* on *f*′(*η*).

**Figure 6:**
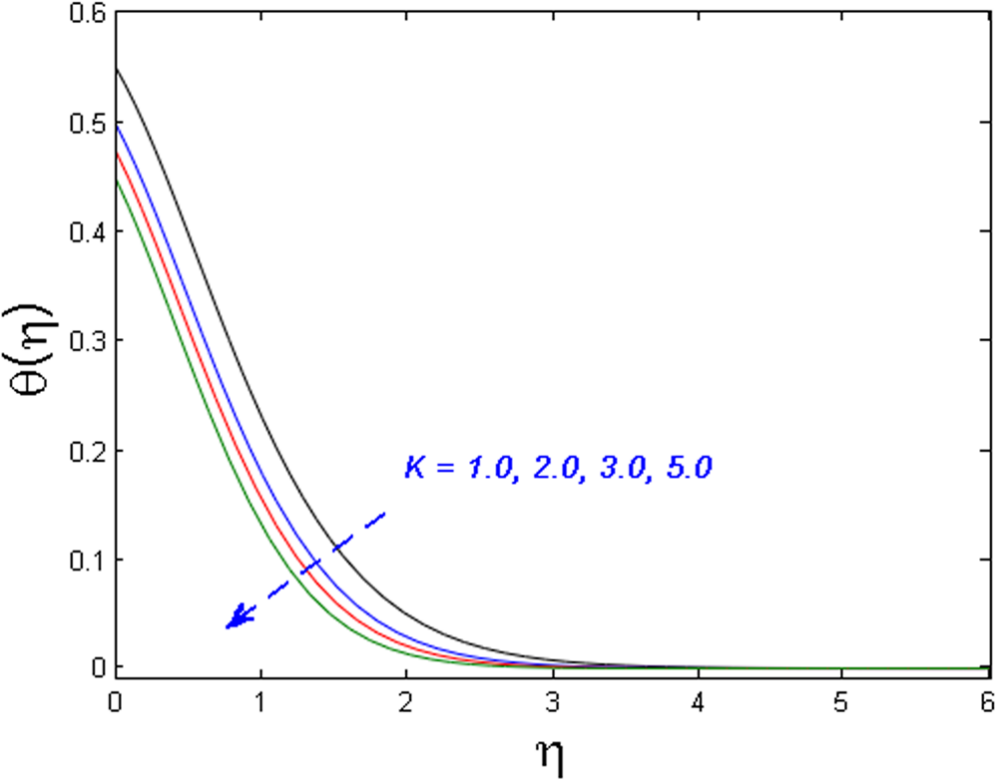
Effect of *K* on *θ*(*η*).

**Figure 7:**
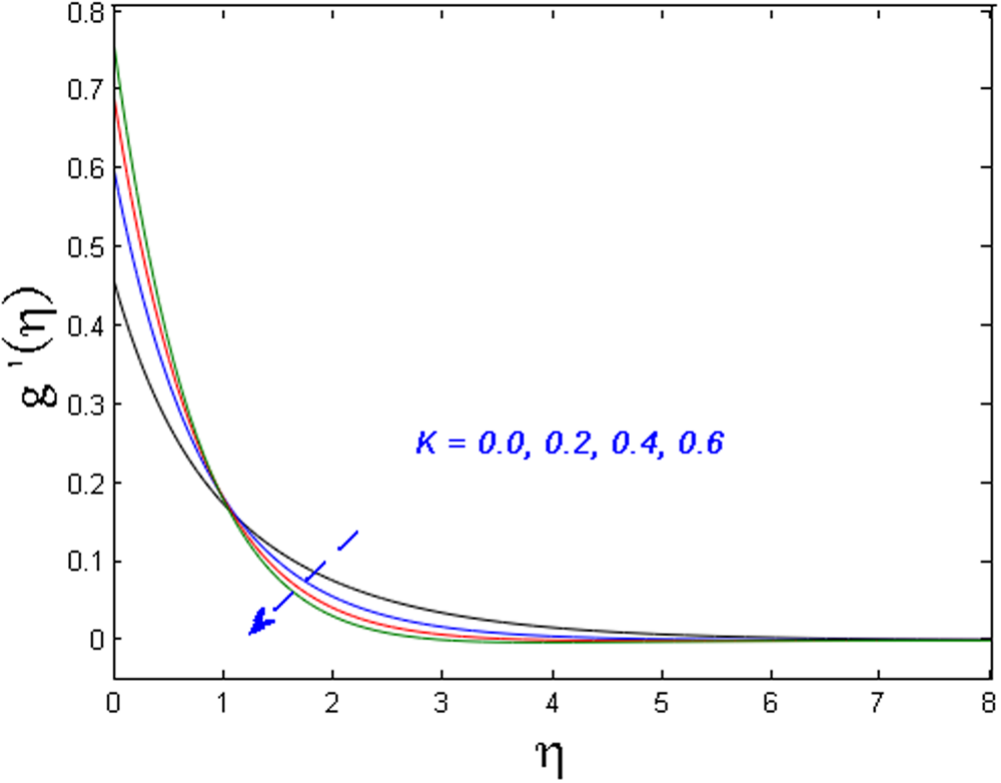
Effect of *K* on *g*′(*η*).

Figures 5–7 examine the effect of micropolar material parameter *K* on axial velocity, microrotation, and temperature profiles. Figure 5 demonstrates the effect of *K* on velocity field. It is witnessed that the velocity field and momentum boundary layer augment versus growing values of the *K*. This is because of the reason that viscosity declines with growing the values of *K*. Due to this low viscosity, the temperature distribution also decreases which is illustrated in Fig. 6. Moreover, the value of microrotation velocity at the wall diminishes continuously from maximum to zero far away from the wall as illustrated in Fig. 7.

**Figure 8:**
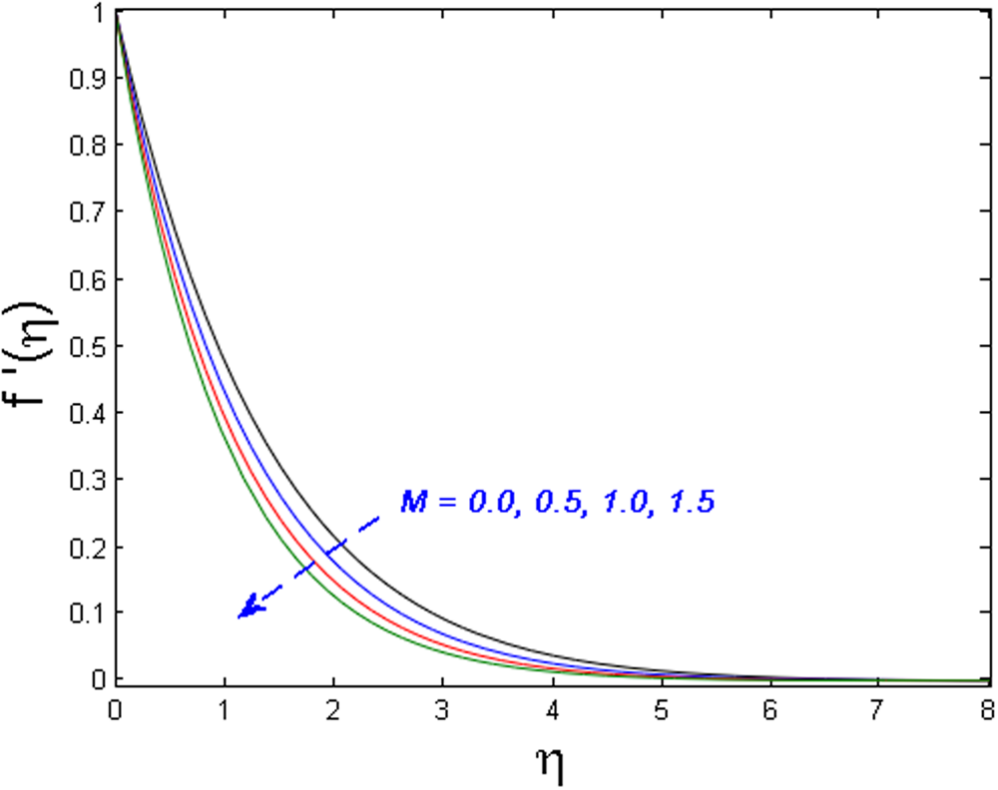
Effect of *M* on *f*′(*η*).

**Figure 9:**
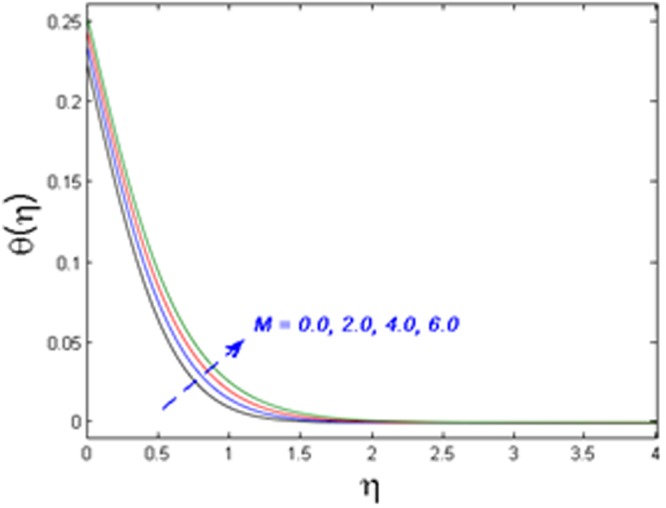
Effect of *M* on *θ*(*η*).

**Figure 10:**
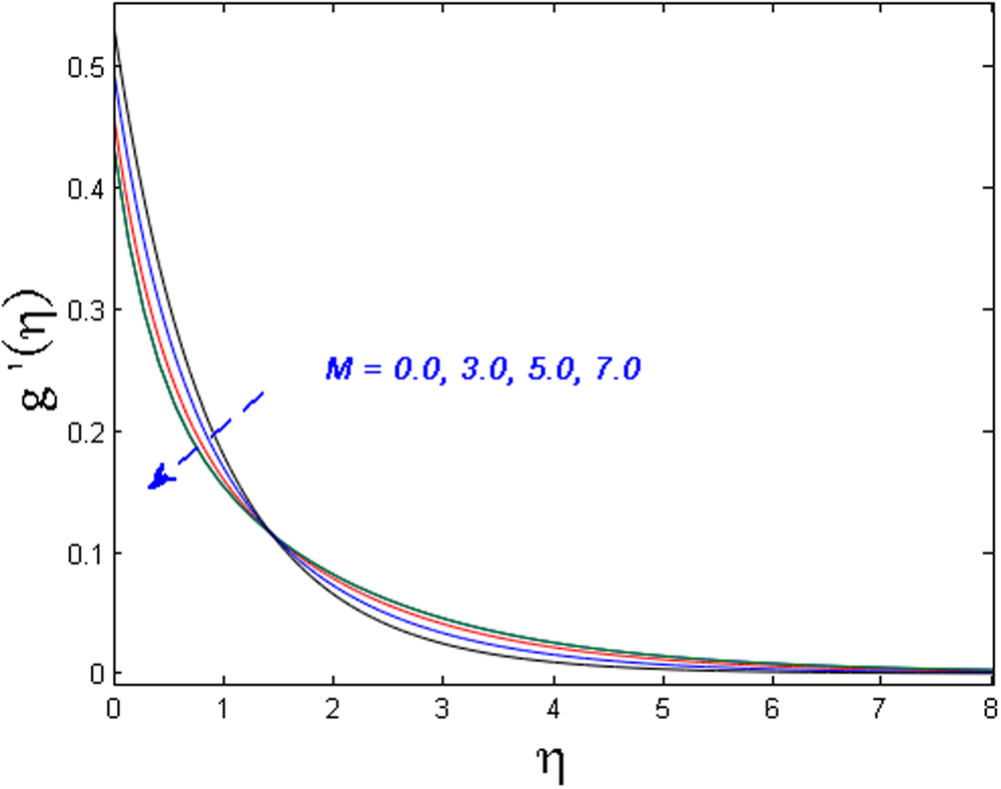
Effect of *M* on *g*′(*η*).

Figures 8–10 outline the behavior of axial velocity, microrotation velocity, and temperature profile for different values of the magnetic parameter *M* respectively. Figures 8 and 9 display the impact of *M* on axial velocity and temperature distribution. It is witnessed that for higher the values of *M*, the velocity profile declines whereas temperature field and thermal boundary layer thickness increase. Actually, the increase in values of M means stronger Lorentz force which offers more resistance to the fluid’s motion. That is why decline in fluid’s velocity is witnessed. Also, because of this fact more heat is produced and as a result, the temperature profile and thermal boundary layer thickness are increased. Figure 10 shows the impact of magnetic parameter *M* on microrotation velocity. For gradually mounting values of magnetic parameter *M*, the microrotation velocity profile diminishes near the boundary and an opposite behavior is observed far-off from the boundary.

**Figure 11:**
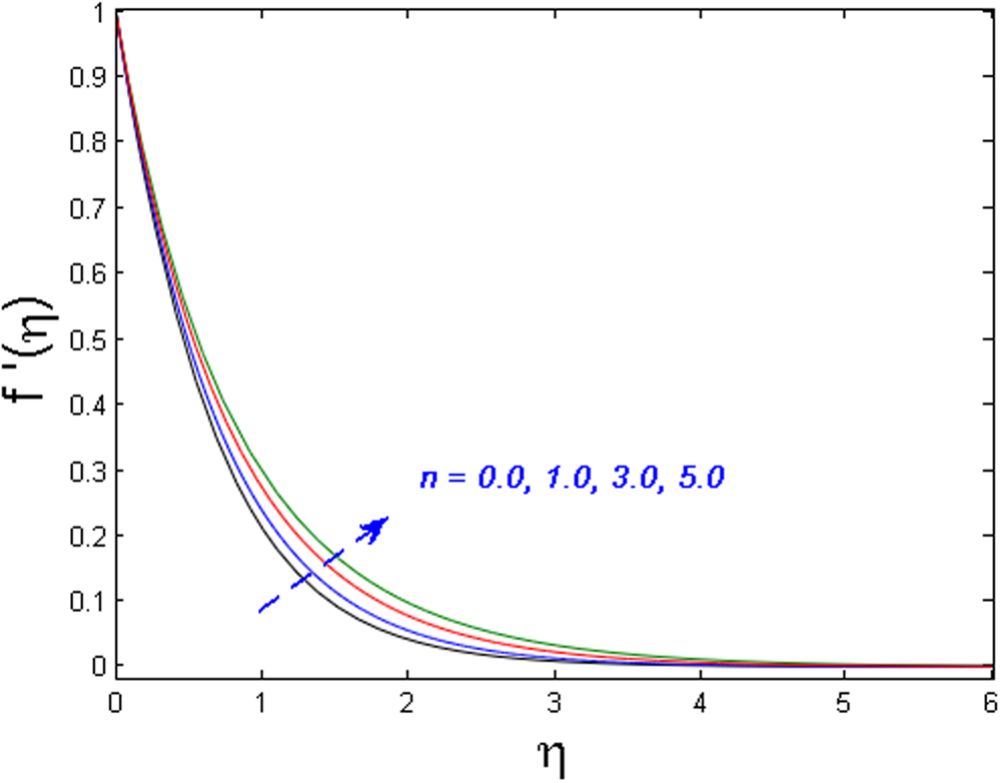
Effect of *n* on *f*′(*η*).

**Figure 12:**
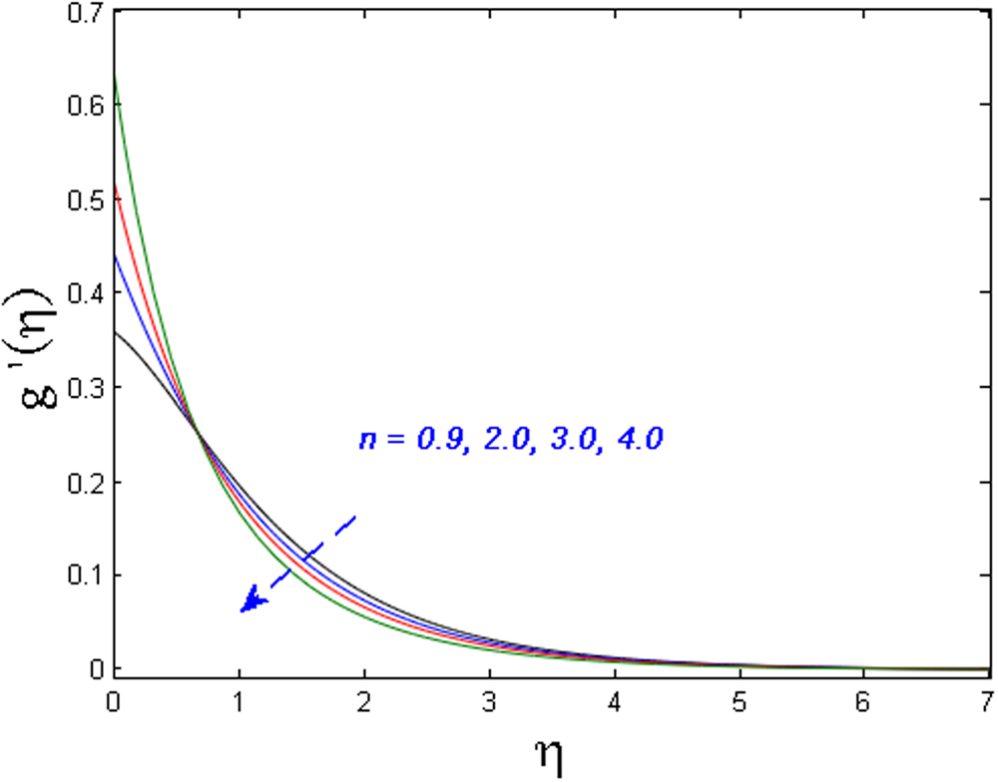
Effect of *n* on *g*′(*η*).

**Figure 13:**
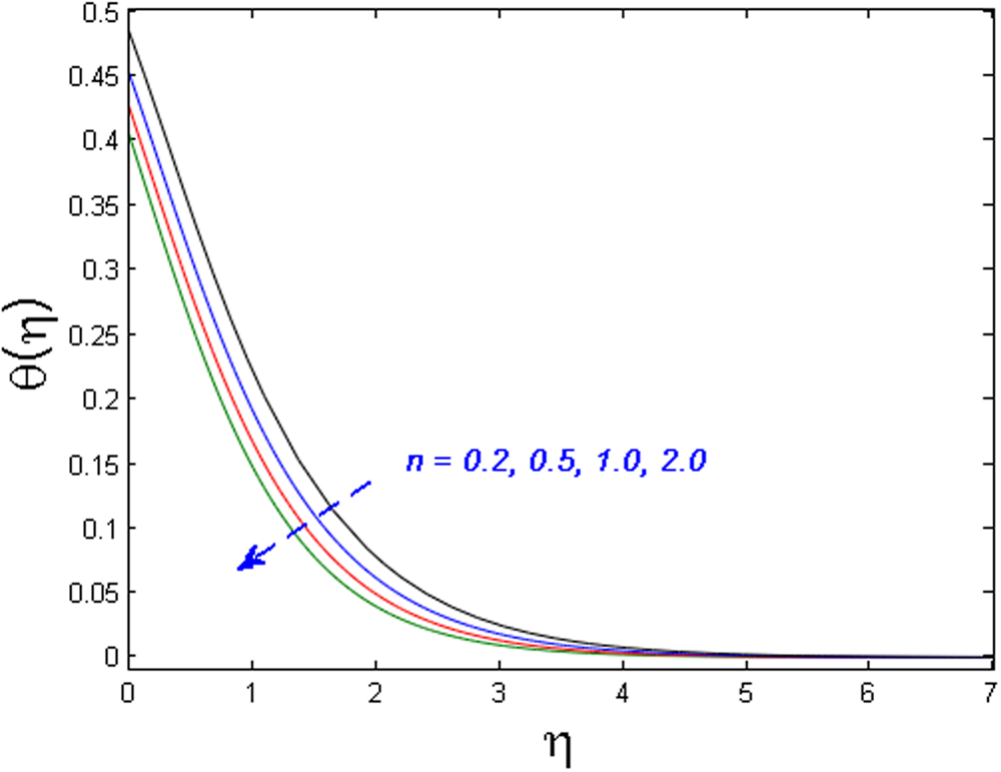
Effect of *n* on *θ*(*η*).

**Figure 14:**
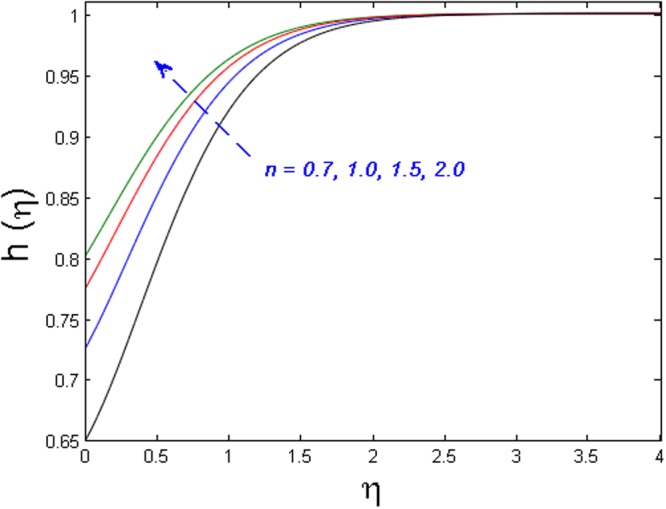
Effect of *n* on *h*(*η*).

Figures 11–14 analyze the axial velocity, microrotation velocity, temperature and concentration fields for numerous values of power-law index parameter *n* respectively. Figure 11 depicts the upshot of *n* on velocity field. It is witnessed that the velocity distribution rises when values of *n* are increased. This is due to the fact that enhanced values of *n* are the main cause for the decrease in the thickness of the wall and as a result, more stretching of the sheet is observed which is responsible for enhanced velocity. However, the microrotation velocity and temperature profiles diminish by enhancing the values of *n* as given in Figs 12 and 13 respectively. Figure 14 illustrates the concentration profile for numerous values of *n*. It is seen that for the higher values of power-law index parameter *n*, the concentration field boosts.

**Figure 15:**
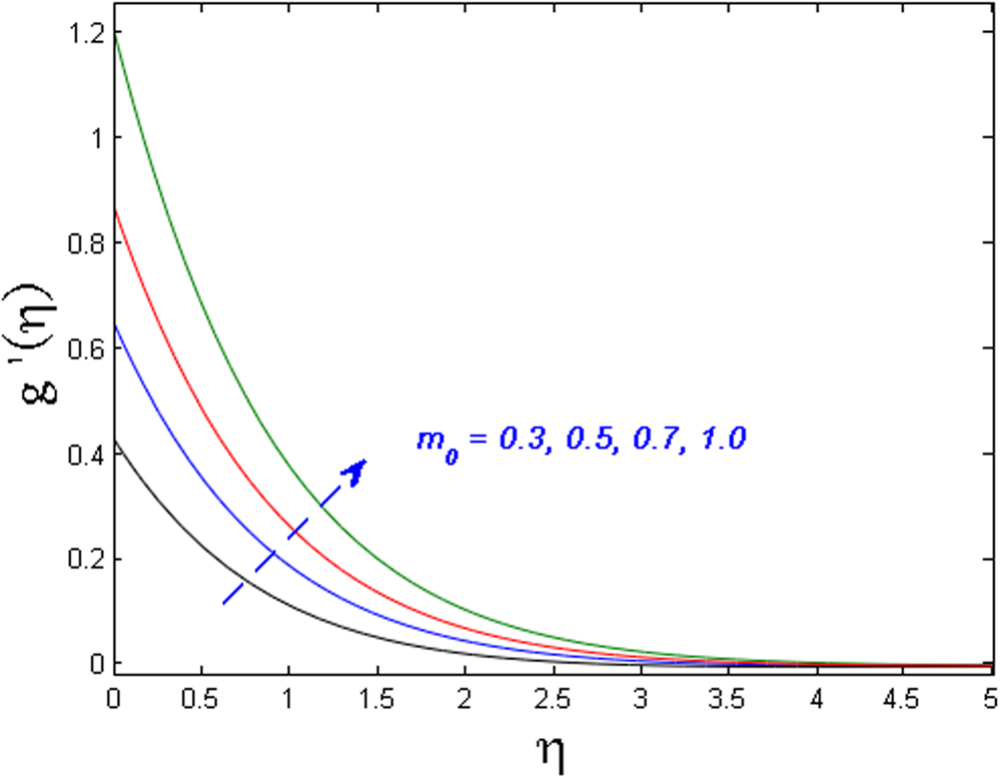
Effect of *m*_*0*_ on *g*′(*η*).

**Figure 16:**
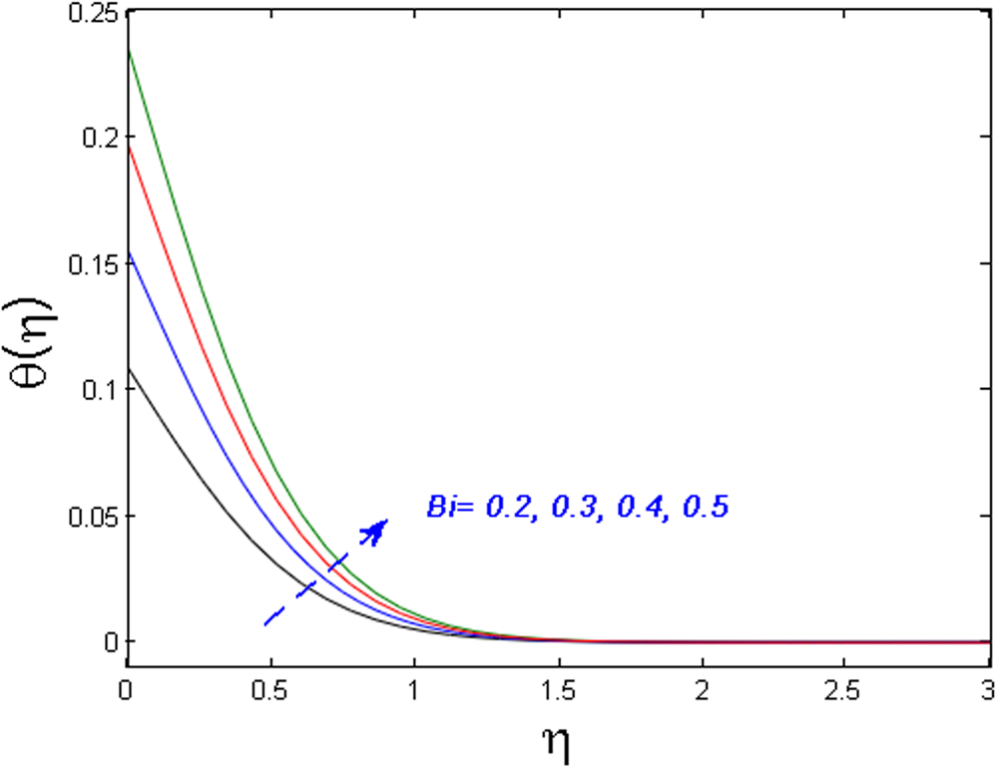
Effect of *B*_*i*_ on *θ*(*η*).

**Figure 17:**
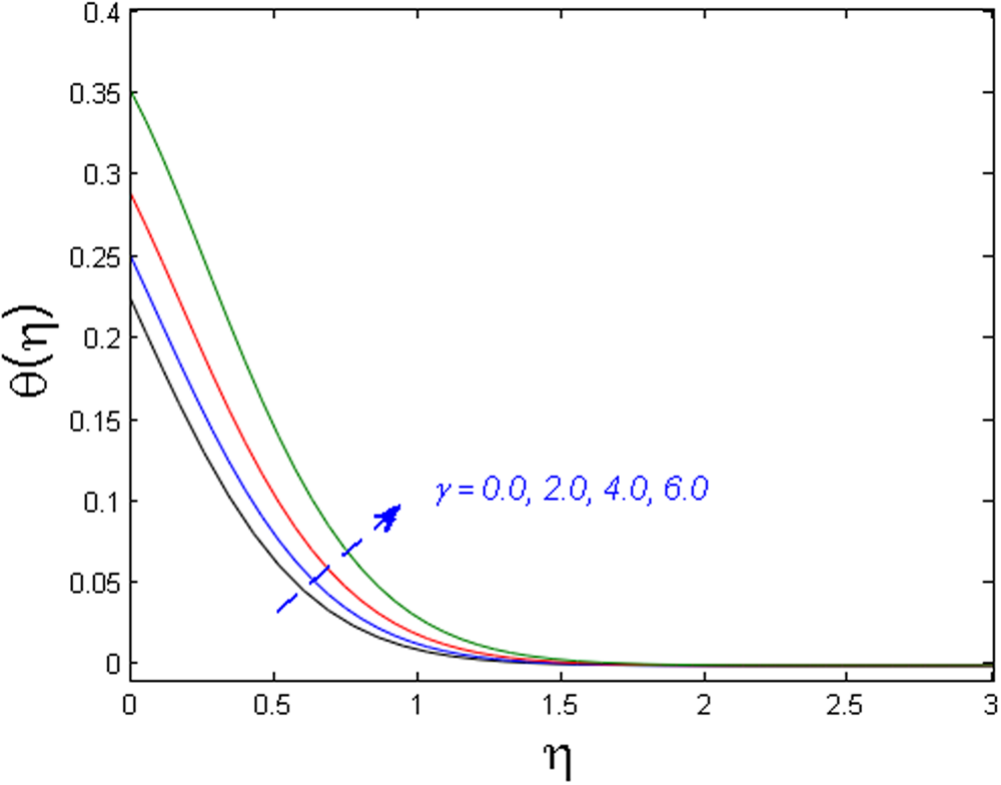
Effect of *γ* on *θ*(*η*).

**Figure 18:**
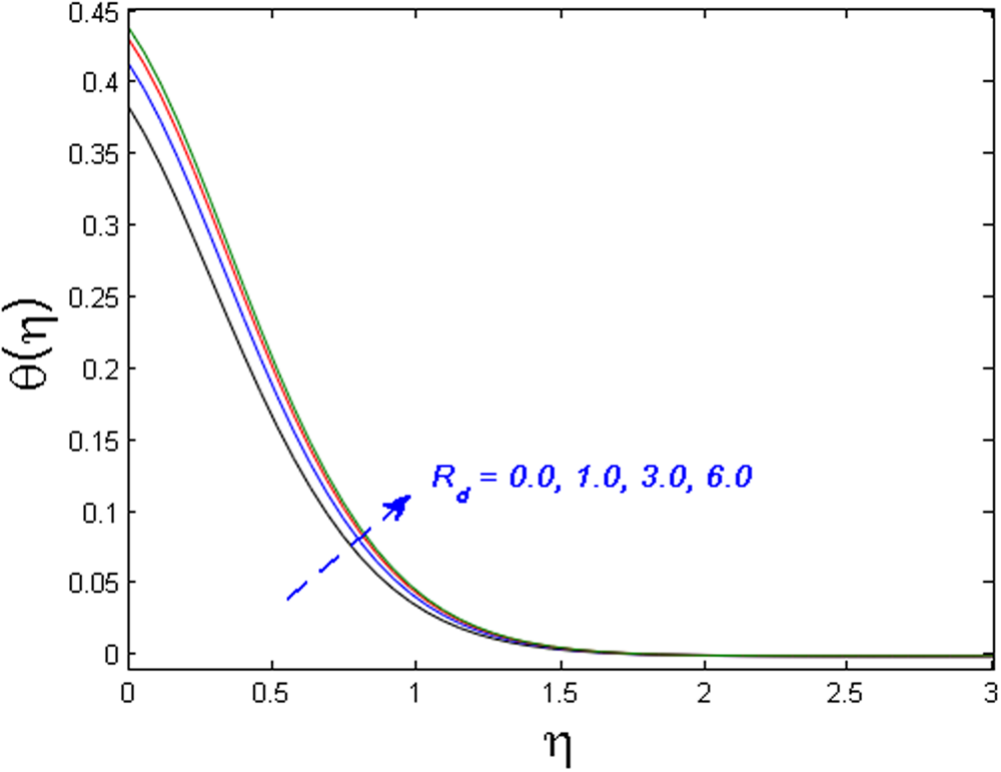
Effect of *R*_*d*_ on *θ*(*η*).

**Figure 19:**
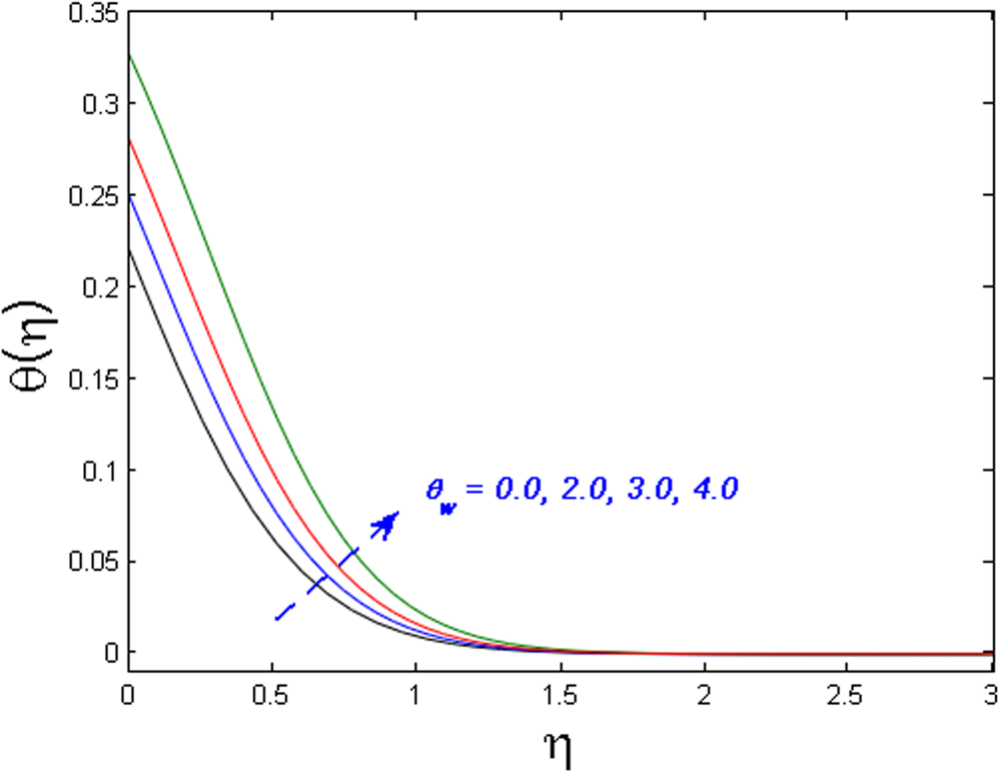
Effect *θ*_*w*_ on *θ*(*η*).

Figure 15 specifies the microrotation velocity field for distinct values of boundary parameter *m*_0._ It is noticed that the microrotation velocity profile is enhanced for growing values of *m*_0_. Figure 16 is graphed to perceive the variation in temperature profile for different values of Biot number *B*_*i*_. It is seen that temperature profile is increased for growing estimations of Biot number. It is due to the fact that increasing values of Biot number *B*_*i*_ rises the heat transfer coefficient which ultimately enhances the temperature. The action of *γ* (heat generation parameter) on the temperature profile is portrayed in Fig. 17. For increasing values of heat generation parameter *γ* means *T*_*w*_ > *T*_∞_ *i.e*., more heat transfer from the surface to the fluid and as a result, the fluid temperature is enhanced. Figures 18 and 19 portrayed to depict the influence of radiation parameter *R*_*d*_ and temperature ratio parameter *θ*_*w*_ on temperature field respectively. It is apparent from the figure that the temperature field is higher for values of both the radiation parameter and temperature ratio parameter. Physically this is due to the fact that with the increase in radiation parameter, the mean absorption coefficient decreases. Hence the rate of radiative heat transfers to the fluid increases.

**Figure 20:**
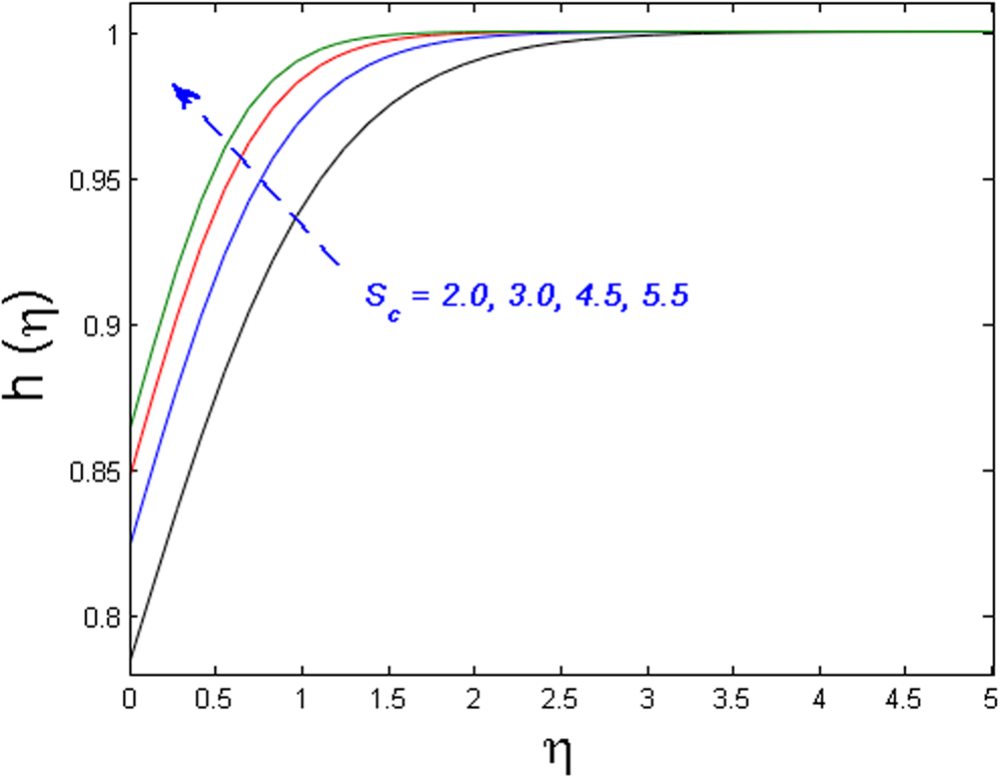
Effect of *S*_*c*_ on *h*(*η*).

**Figure 21:**
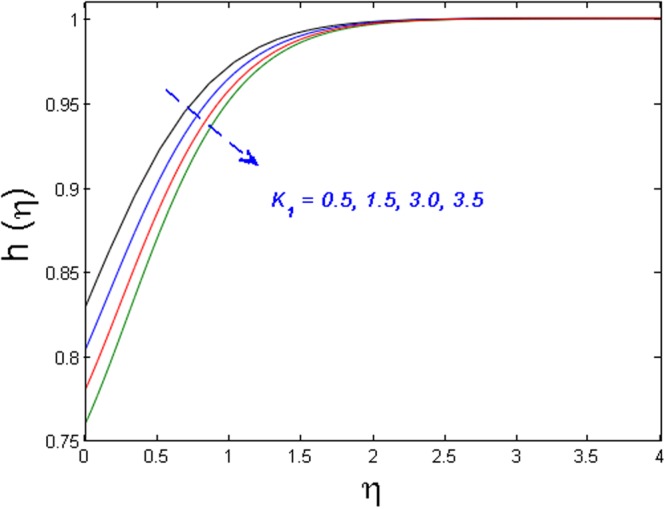
Effect of *K*_1_ on *h*(*η*).

**Figure 22:**
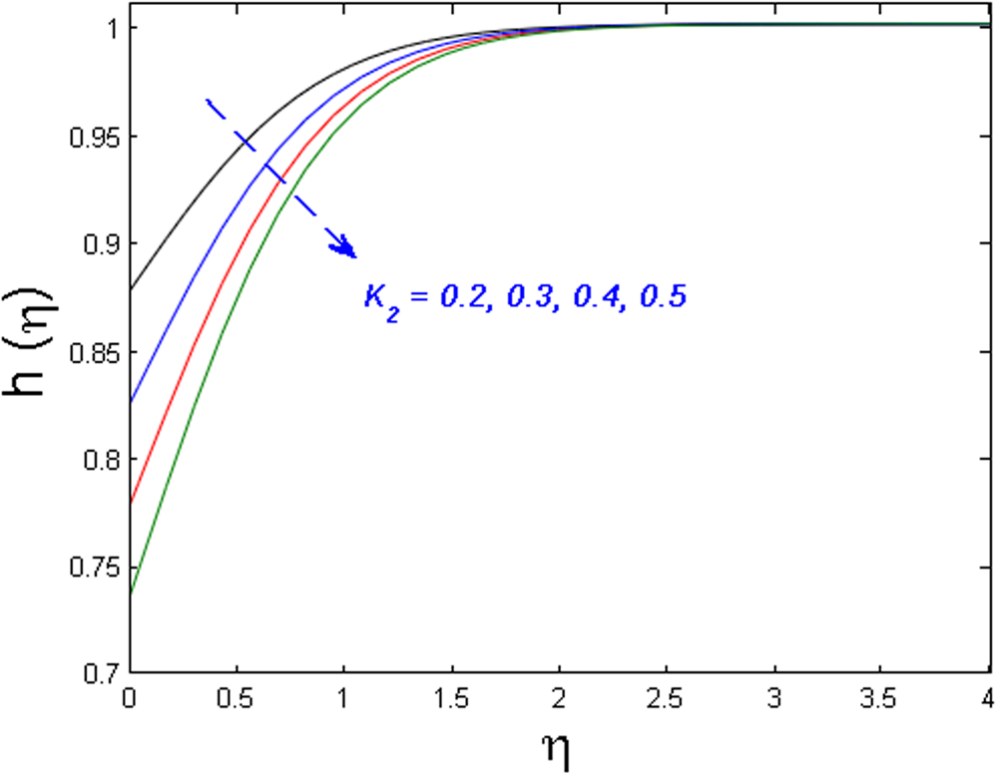
Effect of of *K*_2_ on *h*(*η*).

Figure 20 specifies the concentration profile for varying values of Schmidt number *S*_*c*_. As we know that the Schmidt number is inversely proportional to mass diffusivity. That is why concentration distribution enhances and its corresponding boundary layer thickness diminishes versus gradual increasing values of the *S*_*c*_. In Fig. 21 impact of the strength of homogeneous reaction *k*_1_ on concentration field is described. As the reactants are depleted in a chemical reaction. Because of this fact concentration field shows decreasing the tendency for improving values of *k*_1_. From Fig. 22, it is noticed that concentration distribution is the decreasing function of the strength of heterogeneous reaction *k*_2_. Higher values of *k*_2_ weaken the diffusion coefficient and as a result, less diffused particles abate the concentration field.

**Figure 23:**
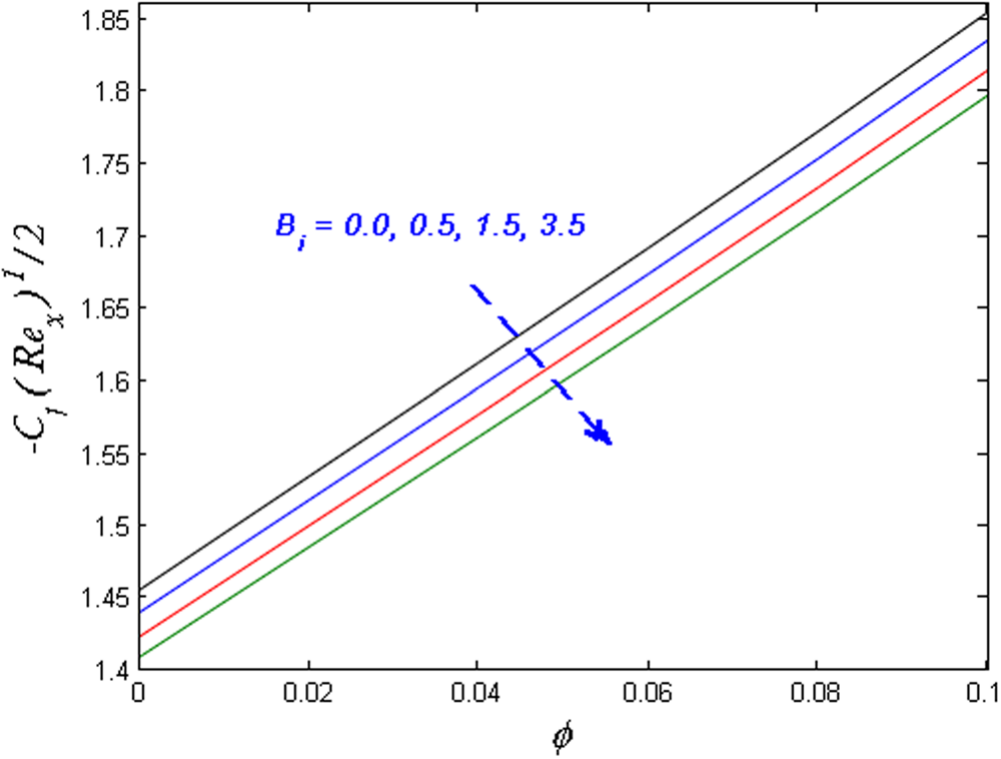
Effects of *ϕ* and *B*_*i*_ on ${{{\rm{Re}}}_{x}}^{1/2}{C}_{f}$.

**Figure 24:**
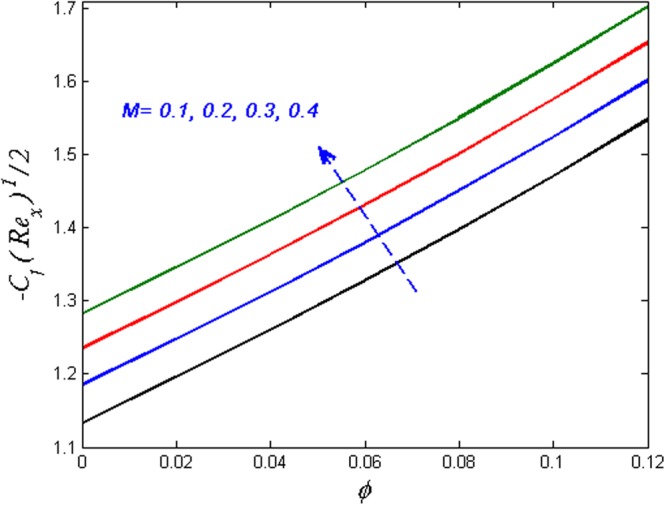
Effects of *ϕ* and *M* on ${{{\rm{Re}}}_{x}}^{1/2}{C}_{f}$.

**Figure 25:**
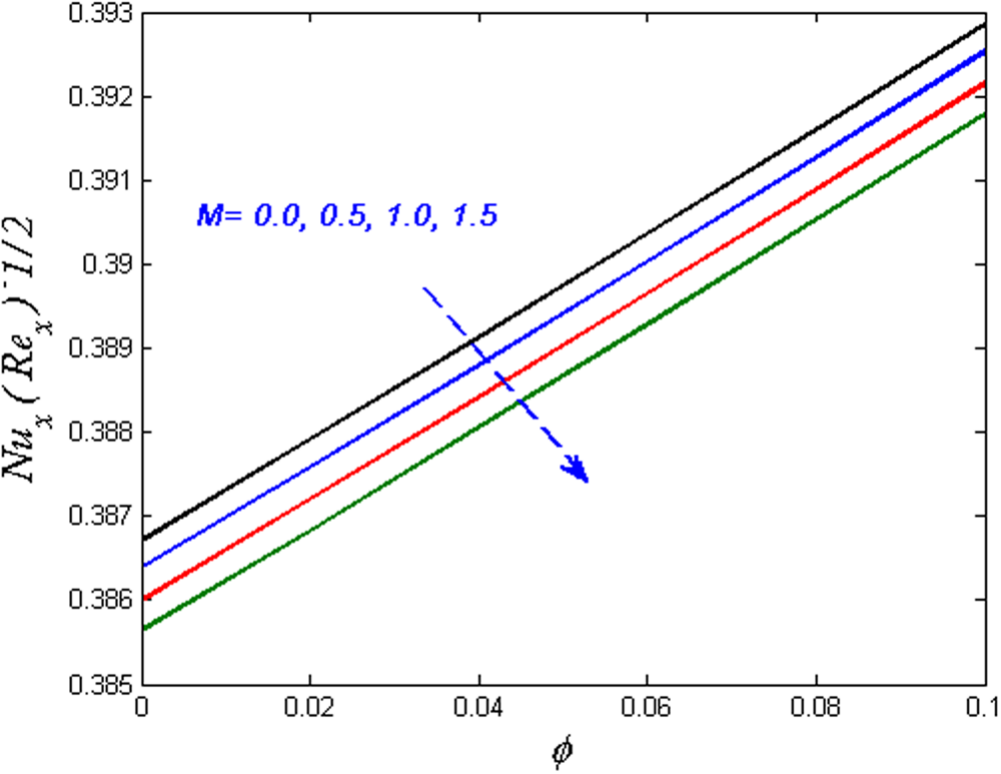
Effects of *ϕ* and *M* on ${{{\rm{Re}}}_{x}}^{-1/2}N{u}_{x}$.

**Figure 26:**
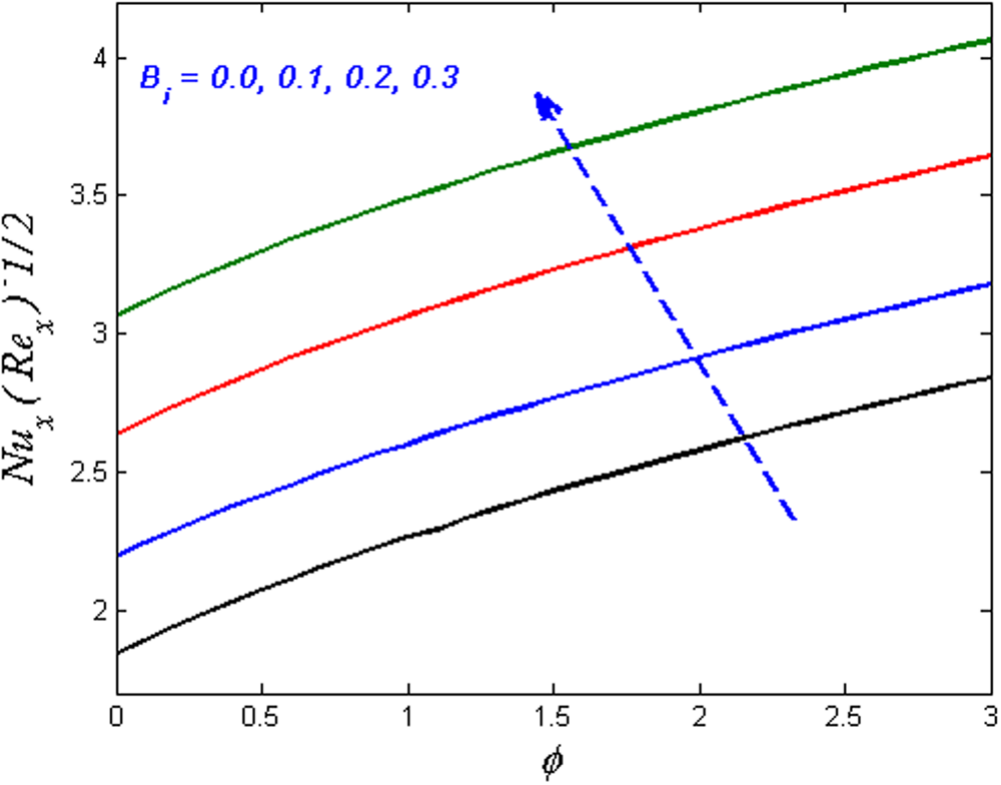
Effects of *ϕ* and *B*_*i*_ on ${{{\rm{Re}}}_{x}}^{-1/2}N{u}_{x}$.

Finally, the effect of physical quantities of interest such as the Skin friction coefficient and the Nusselt number for various values of dimensionless governing flow field parameters are graphed in Figs 23–26 respectively. Figure 23 portrays the impact on Skin friction coefficient ${{{\rm{Re}}}_{x}}^{1/2}{C}_{f}$ for various values of Biot number *B*_*i*_ and solid volume fraction *ϕ*. It can be seen that the Skin friction coefficient decreases versus augmented values of the Biot number *B*_*i*_ and an opposite behavior are observed in case of solid volume fraction *ϕ*. Figure 24 displays effect on the Skin friction coefficient for varied estimated values of the magnetic parameter *M* and solid volume fraction *ϕ*. It is observed that the skin friction coefficient enhances for escalating values of the magnetic parameter and solid volume fraction *ϕ*. Figure 25 demonstrates the effect of the magnetic parameter and the volume fraction on the Nusselt number. It is found that the Nusselt number diminishes with enhancing the value of magnetic parameter however the contradictory impact is observed in the case of volume fraction parameter. Further, the Nusselt number increases with increasing the Biot number and the volume fraction parameter. This effect is displayed in Fig. 26.

Table 4 is erected to display the numerical values of Nusselt number ${{\mathrm{Re}}_{{\rm{x}}}}^{-1/2}N{u}_{x}$ for the varied values of the different parameter. It is seen that Nusselt number increases and Skin friction coefficient decrease for growing values of solid volume fraction *ϕ* and radiation parameter *R*_*d*_. By mounting the values of heat generation parameter (*γ*), the Nusselt number diminishes while it enhances for increasing values of Biot number *B*_*i*_. Moreover, the skin friction coefficient diminishes with enhancing the value of Biot number and it has no effect on skin coefficient with increasing the value of heat generation parameter. Table 5 depicts the behavior of *h*′(0) and *φ*′(0) when *δ* = 1. It is witnessed that for increasing values of *k*_2_, both *h*′(0) and *φ*′(0) show increasing behavior.

**Table 4:** Numerical values of Nusselt number ${{{\rm{Re}}}_{x}}^{-1/2}N{u}_{x}$, skin friction ${{{\rm{Re}}}_{x}}^{1/2}{C}_{f}$ and *g*′(0) when ${\theta }_{w}=1.2,\,{\rm{and}}\,\Pr =6.2$.

*ϕ*	*R*_*d*_	*γ*	*B*_*i*_	${{{\bf{Re}}}_{{\boldsymbol{x}}}}^{{\boldsymbol{-}}{\bf{1}}{\boldsymbol{/}}{\bf{2}}}{\boldsymbol{N}}{{\boldsymbol{u}}}_{{\boldsymbol{x}}}$	${{{\bf{Re}}}_{{\boldsymbol{x}}}}^{{\bf{1}}{\boldsymbol{/}}{\bf{2}}}{{\boldsymbol{C}}}_{{\boldsymbol{f}}}$	*g*′(0)
0.1	03	0.1	0.5	1.70870	1.14570	0.62173
0.2				1.73360	1.11390	0.51651
0.3				1.76080	1.06140	0.42920
0.1	0.3	0.1	0.5	0.53758	1.14640	0.62174
	01			0.84084	1.14600	0.62174
	02			1.27460	1.14580	0.62173
0.1	03	0.1	0.5	1.70870	1.14570	0.62173
		0.3		1.61730	1.14570	0.62164
		0.5		1.38010	1.14570	0.62133
0.1	03	0.1	0.5	1.70870	1.14570	0.62173
			1.0	2.89670	1.13450	0.62164
			1.5	3.74190	1.12700	0.62157

**Table 5:** Numerical values of *h*′(0) and −*φ*′(0) for different values of *k*_2_ when *δ* = 1.

*k*_2_	*h*′(0)	−*φ*′(0)
0.2	0.16977	0.16977
0.3	0.23660	0.23660
0.4	0.29451	0.29451
0.5	0.34515	034515

## Concluding Remarks

In this article, a numerical solution for the effects of h-h reactions on the flow of micropolar nanofluid with water as the base fluid and Ferric oxide as nanoparticles past a nonlinear stretched surface is obtained and discussed. Additional impacts like nonlinear thermal radiation, magnetohydrodynamics, and heat generation/absorption with convective boundary condition are added features towards the novelty of the problem. The transformed system of ordinary differential equations is solved by MATLAB built-in function bvp4 for varied values of arising parameters. The salient points of the present study are given as follows:

- The velocity distribution with its associated boundary layer thickness increase for enhanced values of micropolar parameter *K* while the temperature profile and microrotation velocity diminish.
- For higher values of *k*_1_ and *k*_2_ (strength of homogeneous and heterogeneous reactions), concentration profile reduces.
- For gradually mounting values of magnetic parameter *M*, the microrotation velocity profile contracts near the boundary and opposite trend are witnessed far away from the boundary.
- The Nusselt number and Skin friction coefficient increase and decrease for growing values of solid volume fraction *ϕ* and radiation parameter *R*_*d*_ respectively.
